# Validation of reference genes for gene expression analysis in chicory (*Cichorium intybus*) using quantitative real-time PCR

**DOI:** 10.1186/1471-2199-11-15

**Published:** 2010-02-15

**Authors:** Asad Maroufi, Erik Van Bockstaele, Marc De Loose

**Affiliations:** 1 Department of Plant Production, Faculty of Bioscience Engineering, Coupure Links 653, Gent University, 9000 Ghent, Belgium; 2 Institute for Agricultural and Fisheries Research (ILVO), Burg Van Gansberghelaan 115 bus 1, 9820 Merelbeke, Belgium; 3 Institute for Agricultural and Fisheries Research (ILVO), Burg Van Gansberghelaan 96 bus 1, 9820 Merelbeke, Belgium; 4 Department of Plant Biotechnology and Genetics, Faculty of Sciences, Gent University, KL Ledeganckstraat 35, 9000 Ghent, Belgium

## Abstract

**Background:**

Quantitative real-time reverse transcriptase polymerase chain reaction (qRT-PCR) is a sensitive technique for quantifying gene expression levels. One or more appropriate reference genes must be selected to accurately compare mRNA transcripts across different samples and tissues. Thus far, only actin-2 has been used as a reference gene for qRT-PCR in chicory, and a full comparison of several candidate reference genes in chicory has not yet been reported.

**Results:**

Seven candidate reference genes, including nicotinamide adenine dinucleotide dehydrogenase (*NADHD*), actin (*ACT*), β-tubulin (*TUB*), glyceraldehyde-3-phosphate-dehydrogenase (*GADPH*), histone H3 (*H3*), elongation factor 1-alpha (*EF*) and 18S rRNA (*rRNA*) were selected to study the expression stability for normalisation of gene expression in chicory. Primer specificity and amplification efficiency were verified for each gene. The expression stability of these genes was analysed across chicory root and leaf tissues using geNorm, NormFinder and BestKeeper software. *ACT*, *EF*, and *rRNA *were the most stable genes as identified by the three different analysis methods. In addition, the use of *ACT, EF *and *GAPDH *as reference genes was illustrated by analysing 1-*FEHII *(*FEHII*) expression in chicory root and leaf tissues. These analyses revealed the biological variation in *FEHII *transcript expression among the tissues studied, and between individual plants.

**Conclusions:**

geNorm, NormFinder, and BestKeeper analyses indicated that *ACT*, *EF *and *rRNA *had the highest expression stability across leaf and root tissues, while *GAPDH *and *NADHD *showed relatively low expression stability. The results of this study emphasise the importance of validating reference genes for qRT-PCR analysis in chicory. The use of the most stable reference genes such as *ACT *and *EF *allows accurate normalisation of gene expression in chicory leaf and root tissues.

## Background

Quantitative real-time reverse transcriptase polymerase chain reaction (qRT-PCR) is an efficient, sensitive and reliable technique to quantify transcript expression levels. qRT-PCR is fast, easy to use and provides simultaneous measurement of gene expression in many different samples for a limited number of genes [1,2]. qRT-PCR has various applications, such as clinical diagnostics [3], analysis of tissue-specific gene expression in humans [4], and gene expression studies in plants [5]. Appropriate normalisation is very important for quantification of transcript expression levels. The most accepted approach to quantification is normalisation of the expression level of a gene of interest (target gene) to the expression level of an internal stably expressed gene (control gene) [6-10]. The control gene, often termed reference gene, is a stably expressed gene that is experimentally verified in given species and tissues under given experimental conditions [6]. By normalising the transcript expression level of a target gene to the expression level of a reference gene, differences in the quality or quantity of template RNA and differences in efficiencies of the reverse transcription reaction between samples are accounted for. This allows the direct comparison of normalised transcript expression levels between samples. However, this approach requires the selection of at least one reference gene for validation of a corresponding qRT-PCR method.

This case study illustrates the use of qRT-PCR for improving inulin production in chicory. Chicory (*Cichorium intybus*) is an important crop for inulin production. Inulin is a group of naturally occurring polysaccharides that are produced by many types of plants including Jerusalem artichoke (*Helianthus tuberosus*) and chicory [11]. Inulin is widely used as an ingredient in functional foods, and there is growing interest in food and non-food industries to make new compounds from inulin and its derivatives [12-15]. Currently, chicory is the only plant species used on an industrial scale for the commercial extraction of inulin [15]. Investigation of the expression level of genes encoding enzymes involved in the inulin biosynthesis and degradation pathway will enable breeders to improve inulin content. This case study illustrates the use of qRT-PCR in chicory using the fructan 1-exohydrolaseII a, 1-*FEHIIa *(*FEHIIa*), and fructan 1-exohydrolaseIIb, 1-*FEHIIb *(*FEHIIb*) genes involved in the inulin degradation pathway. The encoded enzymes are believed to catalyse fructan depolymerisation at the end of the growing season, as well as during storage and forcing of tubers and tuberous roots [16-19].

Up to now, Northern blot analysis has been the favoured means of studying the expression of the genes involved in the inulin biosynthesis and degradation pathway [16]. Northern blotting requires a relatively high amount of RNA, and it is laborious and time-consuming [20,21]. Further, in certain cases, the expression of low-expressed genes may be below the detection limit of Northern blotting. A more sensitive and efficient method, such as qRT-PCR, is thus desirable [5]. This requires selection of appropriate reference genes for normalisation. Only actin-2 has been used as a reference gene for qRT-PCR in chicory [22], and a full comparison of several candidate reference genes in chicory has not yet been reported. Taken together, the aims of this study are i) to rank the candidate reference genes according to expression stability across chicory root and leaf tissues using three different methods for expression stability measurements, ii) to develop and evaluate qRT-PCR methods for these genes in chicory, iii) to select appropriate reference genes to use for normalisation of gene expression by qRT-PCR in chicory and iv) to demonstrate their usefulness in qRT-PCR by analysing the expression level of fructan 1-exohydrolaseII, 1-*FEHII *(*FEHII*) in chicory root and leaf tissues, as an example for the genes involved in inulin accumulation.

## Results

### Selection of candidate reference genes and primer design

We selected seven candidate reference genes to validate and develop a qRT-PCR method in chicory, including nicotinamide adenine dinucleotide dehydrogenase *(NADHD) *[23-25], actin (*ACT*) [21,26-31], β-tubulin (*TUB*) [28-33], glyceraldehyde-3-phosphate-dehydrogenase (*GADPH*) [21,26-31,34], histone H3 (*H3*) [34], elongation factor 1-alpha (*EF*) [21,26,27,29,34] and 18S rRNA (*rRNA*) [21,26-30,34]. For all selected genes except *GAPDH*, chicory transcript sequences are available in GenBank to design qRT-PCR primers (Table 1). Based on the *Arabidopsis thaliana GAPDH *sequence (GenBank accession number: AK317337) a primer pair (5'-TGGAGCTGACTTTGTTGTTGA-3'; 5'-TCCACCTCTCCAGTCCTTC-3') was designed that amplified a 298 bp fragment from chicory genomic DNA. This fragment was cloned and sequenced. TBLASTx analysis revealed that this fragment contains an 87 bp region that has at least 89% identity at the amino acid level to *GAPDH *genes across the plant kingdom. We subsequently designed a qRT-PCR primer pair to amplify a 91 bp amplicon covering this region from chicory *GAPDH*.

**Table 1:** Selected candidate reference genes, primers and different parameters derived from qRT-PCR analysis

Gene name	GenBank accession number	Primer sequences (forward/reverse)	Tm (°C)	Amplicon length (bp)	Amplification efficiency (%)	*S.D of efficiency	Average Cp of cDNA	**R^2^
*ACT*	EF528575	CCAAATCCAGCTCATCAGTCG TCTTTCGGCTCCGATGGTGAT	80.26	74	94.31	0.123	24.29	0.9992

*TUB*	AF101419	GCACGGCATTGATGTGACC GAACAAACCTCCCGCCACT	82.56	101	95.77	0.0094	20.65	0.9995

*NADHD*	L39390	TGCAGCAAAGGCTTGTCAAA TCGAAACTTCCCGTTATCCAA	75.84	102	84.89	0.0104	27.12	0.9989

*EF*	AY378166	CATGCGTCAGACGGTTGCTGT CTTCACTCCCTTCTTGGCTGC	82.17	100	98.17	0.0055	19.11	0.9999

*H3*	AY378165	ACAGCTCGCAAATCAACCG GCGGCTTCTTCACTCCACC	83.79	100	94.49	0.0059	26.46	0.9998

*rRNA*	U42501	GGCGACGCATCATTCAAAT TCCGGAATCGAACCCTAAT	80.61	102	91.27	0.0107	7.11	0.9992

*GAPDH*		AGGGCGGTGCTAAGAAAGTCA TCTGGCTTGTATTCCTTCTCATT	81.55	91	91.04	0.0037	29.58	0.9999

*FEHIIa/FEHIIb*	AY323935/AJ295034.1	TAAAGACTTGAAAGAACAAAGTG CGCACCATAACTTGTCGTGTCG	78.98	135	82.24	0.0116	31.53	0.9981

*S.D, standard deviation; **R^2^, correlation coefficient of the slope of the standard curve

Because the nucleotide sequences of chicory *FEHIIa *and *FEHIIb *transcripts are 94% identical [35], it was not possible to design qRT-PCR primers that could differentiate between the two transcripts. We thus designed a primer pair to amplify a specific region of both *FEHIIa *and *FEHIIb *(Table 1 and Additional file 1). The primer pair was designed to cross the exon5/6 junction of *FEHIIa *(Additional file 1), excluding the possibility that amplification occurs from any genomic DNA contamination. In this case, we studied *FEHII *expression as the combined expression of *FEHIIa *and *FEHIIb *transcripts in qRT-PCR.

### Verification of amplicons, primer specificity, Cp data collection and gene-specific PCR amplification efficiency

**Figure 1:**
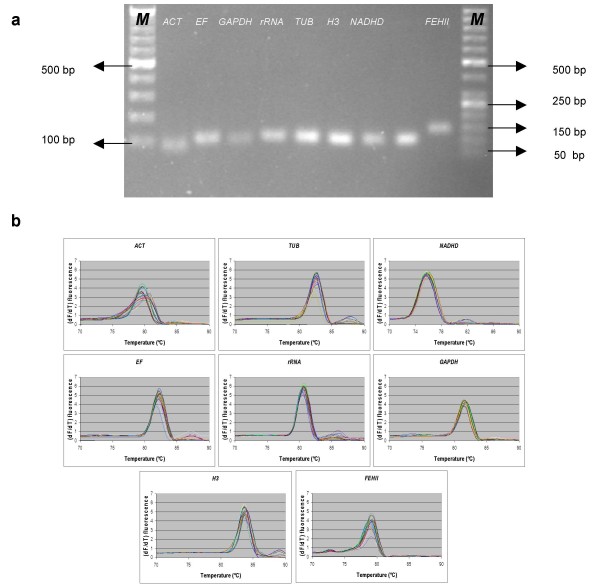
**Confirmation of amplicon size and primer specificity of studied genes**. (a) Agarose gel electrophoresis showing specific reverse transcription PCR products of the expected size for each gene, (b) Melting curves generated for all genes. M represents DNA size marker.

Agarose gel electrophoresis (Figure 1a) and melting curve analysis (Figure 1b and Table 1) revealed that all primer pairs amplified a single PCR product with the expected size. Furthermore, sequence analysis of cloned amplicons revealed that all sequenced amplified fragments were identical or nearly identical (1 bp different for *ACT *and *EF*) to the sequences used for primer design from GenBank, except *H3*, which showed an 18 bp difference in the middle of the amplicon. Real-time RT-PCR was conducted on the 25 samples with eight primer pairs. To reveal the differences in transcript expression levels between studied genes, the average crossing point cycle number (Cp) value was calculated across all leaf and root samples of five individual plants (Table 1). Use of equal amounts of cDNA raised different values from real-time PCR amplification. As expected, the average Cp value varies between the different genes. *rRNA *was the most abundant (mean Cp = 7.11) reference transcript while *GAPDH *was the least abundant (mean Cp = 29.58). A standard curve using a dilution series of the cloned amplicons (spanning five orders of magnitude) was made to calculate the gene-specific PCR efficiency. The correlation coefficient (R^2^) of the slope of the standard curve used to calculate gene specific PCR amplification efficiency (E), and PCR efficiency including standard deviation (S.D), of all selected genes are listed in Table 1. The PCR amplification efficiency for the various PCR methods varied between 82.24% and 98.17% (Table 1).

### Expression stability of candidate reference genes

**Figure 2:**
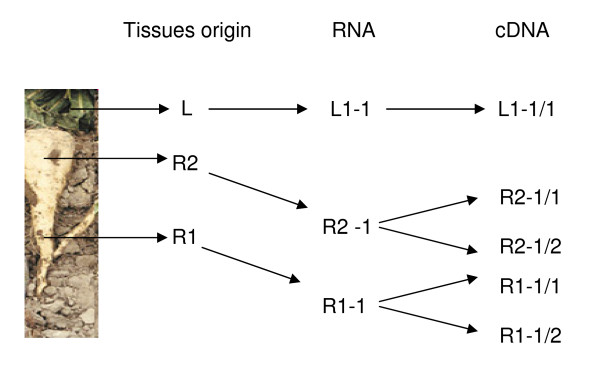
**Schematic diagram illustrating different tissues origin used for RNA extraction and labelling of RNA and cDNA samples used for qRT-PCR**.

Three different software programmes were used to calculate the expression stability of the candidate reference genes: geNorm [36], NormFinder [37] and BestKeeper [38]. To find stably expressed genes, we first assayed gene expression stability across leaf and root tissues (L1-1/1, R1-1/1, R1-1/2, R2-1/1 and R2-1/2 samples; Figure 2). Cp data were collected for all selected tissues of each plant. These data were either used directly for stability calculations (BestKeeper analysis) or were first transformed to relative quantities using the delta-Ct method and the gene-specific amplification efficiency implemented in qBase (geNorm and NormFinder analysis).

#### a) geNorm analysis

**Figure 3:**
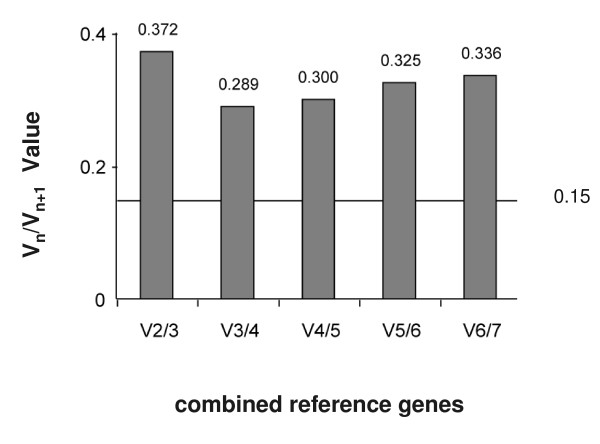
**Determination of the optimal number of reference genes calculated by geNorm**. Determination of the optimal number of reference genes for accurate normalisation of gene expression. Average pairwise variations V_n_/V_n+1 _are calculated between the normalisation factors NF_n _and NF_n+1 _to indicate whether inclusion of extra reference gene adds to the stability of the normalisation factor.

Average expression stability (*M *value) of all genes was calculated by geNorm (version 3.5). The *M *values of the candidate reference genes across chicory leaf and root tissues are shown in Table 2. geNorm recommends using an *M *value below the threshold of 1.5 to identify (sets of) reference genes with stable expression. The three genes *ACT*, *EF*, and *rRNA *had the highest expression stability in leaf and root tissues (the lowest *M *values). *H3 *and *TUB *had intermediary *M *values that are still below the threshold of 1.5. *NADHD *and *GAPDH *had *M *values higher then 1.5, indicating less stable expression across leaf and root tissues (Table 2). To determine the optimal number of reference genes, geNorm calculates the pairwise variation V_*n*_/V_*n*+1_between two sequential normalisation factors NF_*n *_and NF_*n*+1 _that contain an increasing number of reference genes. A large variation means that the added gene has a significant effect on the normalisation and should preferably be included for calculation of a reliable normalisation factor. Ideally, extra reference genes are included until the variation V_*n*_/V_*n*+1 _drops below a given threshold. Vandesompele and colleagues recommended a threshold of 0.15, although this threshold should not be viewed as too strict of a cut-off [36]. In our data sets, the calculated NF_*n *_at increasing numbers of reference genes shows that the inclusion of the third, fourth and fifth genes (*rRNA*, *H3 *and *TUB; M *values < 1.5) still contribute significantly to the variation of the normalisation factor (V_2/3_, V_3/4 _and V_4/5 _> 0.15). *NADHD *and *GAPDH *had *M *values higher then 1.5, suggesting that they should not be included in the normalisation factor (Figure 3).

**Table 2:** Ranking of the candidate reference genes according to their stability value using geNorm, NormFinder and BestKeeper analysis

Gene name	Stability value (geNorm)	Ranking order (geNorm)	Stability value (NormFinder)	Ranking order (NormFinder)	Stability value (BestKeeper)	Ranking order (BestKeeper)
*ACT*	0.37	1	0.127	1	0.866	2

*EF*	0.37	1	0.193	2	0.847	3

*rRNA*	0.88	2	0.509	3	0.948	1

*H3*	1.08	3	0.684	4	0.479	6

*NADHD*	1.55	5	1.296	6	-0.069	7

*TUB*	1.30	4	1.243	5	0.759	4

*GAPDH*	1.84	6	1.598	7	0.635	5

#### b) NormFinder analysis

The stability value of each gene was calculated by NormFinder (Table 2). Genes that are more stably expressed are indicated by lower average expression stability values. The analysis ranks *ACT, EF, rRNA *and *H3 *as the four most stable genes (Table 2). Thus, both geNorm and NormFinder rank the same four genes as the most stable and the entire order is identical. Both analyses rank *GAPDH *as the least stable gene.

#### c) BestKeeper analysis

BestKeeper analysis determines the most stably expressed genes based on the coefficient of correlation (r) to the BestKeeper Index (BI), which is the geometric mean of Cp values of candidate reference genes. Variations of gene expression, displayed as the standard deviation of the Cp values, were determined. BestKeeper analysis revealed that *H3 *is the gene with the lowest overall variation, and *GAPDH *with the highest, from the list of selected genes with an S.D of 0.74 and 2.29, respectively (Table 3). The stability value of individual genes was calculated by BestKeeper based on the pairwise correlation between genes and BI (Table 2). The BestKeeper revealed that the best correlations were obtained for *rRNA *(r = 0.948), *ACT *(r = 0.866), *EF *(r = 0.847) and *TUB *(r = 0.759) with p value of 0.001 (Table 3). *GAPDH *is ranked as the fifth stable gene but it has the highest S.D. *H3 *and *NADHD *are ranked as the least stable genes.

**Table 3:** Statistics results by BestKeeper software for seven selected genes based on Cp values

	*H3*	*EF*	*ACT*	*NADH*	*rRNA*	*TUB*	*GAPDH*	BI
n	15	15	15	15	15	15	15	15

GM (Cp)	26.59	19.29	24.39	26.65	7.09	21.12	29.24	20.38

S.D (± Cp)	0.74	1.10	1.11	1.22	1.24	2.14	2.29	1.16

CV (%Cp)	2.79	5.67	4.53	4.59	17.10	10.09	7.81	5.66

n, number of samples; Cp, Crossing point cycle number equivalent terminology for Ct; GM(Cp), the geometric mean of Cp; S.D (± Cp), Cp standard deviation; CV (%Cp), variance coefficient expressed as percentage of Cp level; BI, BestKeeper Index.

In conclusion, the three most stable reference genes commonly identified by the three different analysis methods are *ACT*, *EF*, and *rRNA*. *GAPDH *ranks fifth according to BestKeeper, or seventh according to geNorm and NormFinder.

### Evaluation of expression ratios of candidate reference genes

**Figure 4:**
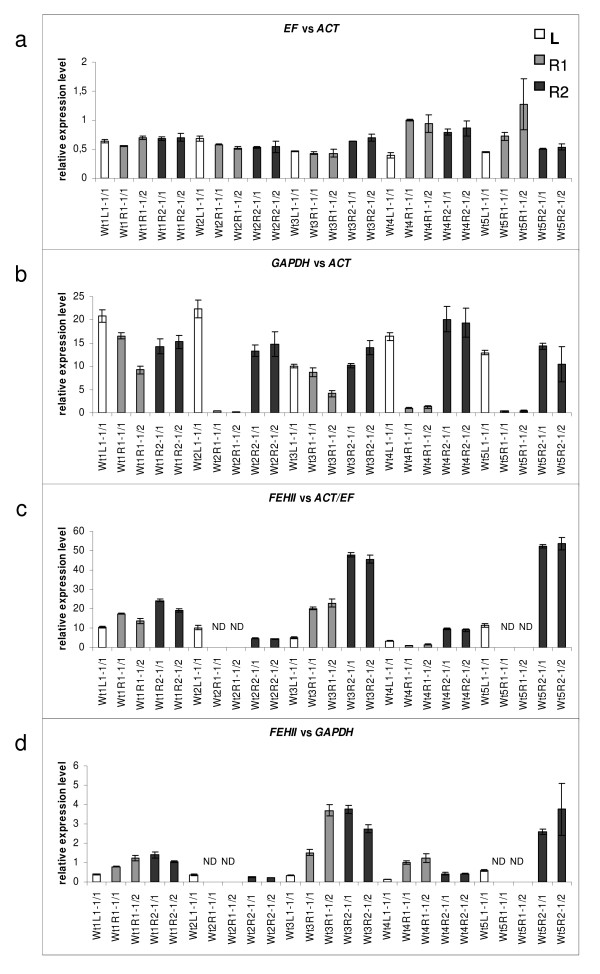
**The relative expression level of EF, GAPDH and FEHII in chicory leaf and root**. (a) *EF *normalised by *ACT*, (b) *GAPDH *normalised by *ACT*, (c) *FEHII *normalised by combined normalisation factor using *ACT *and *EF *and (d) *FEHII *normalised by *GAPDH *in young green leaf (L) and root tissues (R) of five wild type (Wt) chicory plants. Data are obtained from two independent RNA extractions for leaf tissue and two independent cDNA syntheses for R1 and R2 tissues. Expression profiles are calculated in qBase using selected reference genes and the gene specific amplification efficiency. Wt4-R1-1 as calibrator, ND: *FEHII *transcript was not detected in respective samples. Error bars represent standard error of the mean (white bars L samples, gray bars R1 samples and black R2 samples).

The *EF *and *GAPDH *were respectively selected as stable and relatively unstable reference genes to show their expression ratios when normalised by *ACT *in 25 samples including L1-1/1, R1-1/1, R1-1/2, R2-1/1 and R2-1/2 cDNAs (Figure 4a and Figure 4b). The expression pattern of *EF *normalised to *ACT *illustrates the relatively stable expression ratio of these genes across the leaf and root tissues (Figure 4a). The expression patterns of *GAPDH *normalised against *ACT *show that the expression levels are more variable (Figure 4b). In particular, these results reveal the relatively low level of *GAPDH *expression in the R1 samples in three out of five independent plants. This illustrates the reduced expression stability of the *GAPDH *gene in the sample set.

### Application of the qRT-PCR protocol to evaluate the expression of fructan exohydrolase II in chicory leaf and root tissues

To demonstrate the usefulness of the validated candidate reference genes in qRT-PCR, we analysed the expression of *FEHII *across leaf and root tissues (25 samples of five individual plants from cultivar 'Hera' including L1-1/1, R1-1/1, R1-1/2, R2-1/1 and R2-1/2 cDNAs). The relative expression level of *FEHII *in leaf and root tissues was calculated in qBase using the two best reference genes (*ACT *and *EF*) for normalisation (Figure 4c). Expression analysis shows that, in general, *FEHII *transcript expression was detected in all tissues in the five individual plants investigated, except for four cases (Figure 4c). As expected, the highest levels of *FEHII *expression were observed in the mature root tissues (Wt1R2, Wt3R2 and Wt5R2). *FEHII *was generally expressed at a very low level in green leaves (L) and root tips (R1; Figure 4c). Among the leaf samples, the highest-fold change in expression is 3.52 between Wt5L1-1/1 and Wt4L1-1/1 and the lowest-fold change is 1.5 between Wt3L1-1/1 and Wt4L1-1/1. Among the R2 tissues, the highest-fold change is 12.33 between Wt5R2-1/2 and Wt2R2-1/2 and the lowest-fold change is 1.91 between Wt4R2-1/2 and Wt2R2-1/1. These analyses show the biological variation in *FEHII *transcript expression among the studied tissues and between individual plants of the same cultivar. These expression results are consistent with *FEHII *transcript analysis by Northern blot as reported by Van den Ende and colleagues [16], who demonstrated that the expression of *FEHII *was highly abundant in roots. The current study also suggests that qRT-PCR analysis makes it possible to detect and quantify low levels of *FEHII *expression in leaf tissue.

Next, we examined the effect of choosing a reference gene with relatively low expression stability across the target tissues. To this end, the expression of *FEHII *using *GAPDH *as a reference gene was calculated. In comparison with normalisation of *FEHII *data against *ACT/EF *the overall expression profile appears similar (Figure 4c and 4d), but marked changes are introduced in individual samples. For instance, relatively low expression of *GAPDH *in R1 tissues of Wt2, Wt4 and Wt5 (Figure 4d) leads to overestimation of *FEHII *expression in the respective samples. In Wt4, the R1 sample has the lowest *FEHII *expression compared to L and R2, if expression is normalised using *ACT *and *EF*. In contrast, *FEHII *expression in the R1 sample appears much higher than L and R2 if expression is normalised against *GAPDH*. A similarly strong apparent increase is observed for the R1 samples in Wt3 and Wt5 when expression is normalised against *GAPDH*.

Reproducibility of the cDNA synthesis (R1 and R2 samples) was assessed using the best normalisation factor (combination of *ACT *and *EF*; Figure 4c). The similarity between the two observations of each root sample per individual plant reveals high reproducibility between independent cDNA syntheses for the root tissues. In contrast, reproducibility appears to be much lower, especially in the R1 samples, when *FEHII *expression is normalised against *GAPDH*, as compared to normalisation against *ACT *and *EF*. This effect is clearly due to low stability of *GAPDH *expression across samples. This illustrates the adverse effect of an unsuitable reference gene.

## Discussion

qRT-PCR has become a valuable tool for accurate gene expression profiling in addition to Northern blotting [5,39,40]. qRT-PCR is a rapid, accurate and sensitive technique for relative quantification of transcript expression levels and requires a relatively low amount of RNA. Quantification of gene expression is affected by several factors, including experimental sources of variation and the normalisation method. Various experimental sources of variation exist in qRT-PCR, such as sample-to-sample variation in RNA integrity, differences in reverse transcriptase reaction efficiency and the amount of cDNA template used in each PCR reaction. Normalisation of the expression level of a target gene against a stably expressed internal gene can compensate for all these kinds of variations and results in the relative quantification of gene expression levels across samples [41]. Moreover, correct and accurate sample normalisation is required to reveal small but significant differences in expression when comparing samples from different organs or tissues. The accuracy of the results obtained by qRT-PCR therefore strongly depends on the choice of one, or preferably more, reference genes that are stably expressed across all tissues or organ samples [7,8].

Some genes, such as *ACT*, ubiquitin 10 (*UBQ10*), glucose-6-phosphate dehydrogenase (*G6PD*), *GAPDH*, ribosomal genes, cyclophilin, *EF *andalpha-tubulin (*TUA*) are commonly used housekeeping genes for gene expression studies in many plant species [21,26,27,29]. However, recent studies indicate that the traditional housekeeping genes are not always stably expressed when tested in other species or in a wider range of experimental treatments [42-44]. For example, Nicot and colleagues demonstrated that *ACT *did not appear to be the best gene to use as reference gene during the different treatments [31]. In addition, Gutierrez and colleagues have found high variability in the relative expression of common reference genes, including *ACT*, *TUB, UBQ *and *EF*, during various developmental stages in *Arabidopsis *[10].

This means that the most stable reference gene(s) should be identified for a specific species under study or in a new experimental set-up. Accordingly, for gene expression studies in chicory, the stability of reference genes needs to be verified prior to use in qRT-PCR. Actin-2 is the only reported gene used in qRT-PCR in chicory [22]. The direct transfer of traditional and recently proposed novel candidate reference genes by Czechowski and colleagues [34] to non-model plants such as chicory is hampered by the limited availability of genomic sequences. We thus selected a series of candidate reference genes for which such sequence information was available. We developed a qRT-PCR method for *ACT*, *EF*, *rRNA*, *GAPDH*, *H3*, *TUB*, *NADHD *and *FEHII *as the target gene. The specificity of the qRT-PCR primer pairs was confirmed by agarose gel electrophoresis, Tm analysis and sequencing of the amplicons. The PCR amplification efficiency was estimated, and the reference genes were ranked according to their expression level stability across chicory leaf and root tissues using three different methods.

We used geNorm, which has been recently noted as one of the best methods to determine the most stably expressed genes for qRT-PCR analysis [10,42-44]. In addition, geNorm supplies more information about the optimal number of genes in a given experimental dataset. The analysis showed that the expression of *ACT *and *EF *are the most stable across tissues as compared with the other selected genes. Data analysis of expression stability (*M *value) and normalisation factor variation (V_*n*_/V_*n*+1_) determined that *rRNA*, *H3 *and *TUB *can be added in combination with *ACT/EF *to calculate a normalisation factor based on multiple reference genes [7]. The stability of a candidate gene is determined by pairwise comparison of variation of expression ratio in geNorm. In order to avoid co-regulation, following the lead of many other reports [44-46], we also determined the stability of the selected genes using Normfinder, which is less sensitive to co-regulation of the reference genes. NormFinder identified *ACT, EF*, *rRNA *and *H3 *as the four most stable genes, which supports the geNorm analysis in this experiment. According to the results obtained from BestKeeper analysis ranking of the five most stable genes was as follows: *rRNA, ACT, EF, TUB *and *GAPDH*. Pfaffl and colleagues demonstrated that low-expressed genes (*GAPDH *in our dataset; Table 1), with Cp values around cycles 30-35, definitely show different variance compared to high-expressed genes (*rRNA*) with Cp values around 15 or even less. Such two genes can only be correlated on their ranking, not parametrically [41]. Thus, when comparing genes with very different expression levels, it is necessary to use a new model-based analysis that also employs non-parametric methods such as the Spearman and Kendall Tau correlation coefficient. Nevertheless, the latter algorithm is affected by a circular statistical problem; among these three algorithms Bestkeeper is inferior to the two other algorithms. Recently, other studies on validation of reference genes have shown that *GAPDH *is not stable in different tissues or environmental conditions [6,44,47]. Czechowski and colleagues [34] compared traditional and novel reference genes in *Arabidopsis *and found that *GAPDH *was ranked among the 100 most stably expressed genes only after omission of seed and pollen samples, while *TUB6*, *EF-1*α and *ACT2 *were never represented in the top 100. These results also indicate that there are no universal reference genes for all plant species. Validation is thus essential for any selected housekeeping gene used as reference gene in gene expression analysis. In conclusion, the three algorithms do not rank the candidate reference genes in the same order, but all indicated that *rRNA*, *ACT *and *EF *are the most stably expressed genes, given the experimental conditions applied in this study. *H3*, *TUB*, *GAPDH *and *NADHD *were ranked differently by different software programmes' analysis.

*rRNA *is one of the highly stably expressed genes (Table 2). However, there are some drawbacks when using this gene as reference. One of the main problems of using total RNA for normalisation is the large quantity of 18S or 28S *rRNA *transcripts in comparison to the target mRNA transcripts [48], as revealed by a relatively low Cp value for *rRNA *in this experiment. Additionally, the mRNA fraction in total RNA may differ from sample to sample [48]. Due to the high abundance of 18S and 28S *rRNA *transcripts, when using *rRNA *as an internal control for quantification of genes with relatively low expression levels (such as *FEHII*), the cDNA templates may need additional dilution to improve comparison.

Increasing the number of reference genes for normalisation will improve the accuracy of the analysis, but it is expensive and time-consuming. Use of two stable reference genes is a valid normalisation strategy in most cases, and has already resulted in more accurate and reliable normalisation compared to the use of a single reference gene [36]. The current study suggests that *ACT *and *EF *would be two valid reference genes for gene expression study in chicory leaf and root tissues. The qRT-PCR methods described here have important applications in quantifying gene expression levels in chicory as shown by the *FEHII *expression analysis. This analysis showed that the *FEHII *transcript in different plant and tissue samples varies widely. The relative expression analysis of *FEHII *also showed that the expression level is low, but still detectable, in leaf (L) and root tip tissues (R1) and is more highly expressed in mature root tissue (R2). We conclude that the qRT-PCR methods described here facilitate sensitive and accurate quantification of gene expression in chicory.

## Conclusion

To the best of our knowledge, this article is the first attempt to validate a set of commonly used candidate reference genes in chicory for the normalisation of gene expression analysis using qRT-PCR. Analysis of stability using geNorm, NormFinder and BestKeeper revealed that the expression of *ACT *and *EF *is the most stable across root and leaf tissues. In addition, data analysis using geNorm suggested that three genes (*rRNA*, *H3 *and *TUB*) can be added in combination with *ACT/EF *to calculate the normalisation factor based on multiple reference genes [7]. The expression analyses of *FEHII *emphasise the importance of validating reference genes to achieve accurate qRT-PCR analysis. These methods may further be employed to identify the most stable reference genes in other tissues or under other experimental conditions in future studies on chicory.

## Methods

### Plant materials

We sowed seeds of the synthetic root chicory cultivar 'Hera', commonly used for commercial inulin production, then transferred seedlings to pots containing soil and grown under standard conditions in a greenhouse. Leaf (L) and root tissue samples were collected from five plants once the mature root developed (upper part of root (R2) and lower part of root (R1); Figure 2). The tissue samples were immediately frozen in liquid nitrogen. The R2 samples represent the part of the mature root that is normally used for inulin extraction.

### Total RNA isolation and first strand cDNA synthesis

Total RNA was isolated using the RNeasy Plant Mini Kit (Qiagen). For all tissues a single RNA extraction was performed (RNA samples L1-1, R1-1 and R2-1; Figure 2). Total RNA concentration and purity was determined using a Nanodrop ND1000 spectrophotometer (Thermo SCIENTIFIC). Total RNAs were treated with TURBO™ DNase (Ambion) according to the manufacturer's instructions to remove all genomic DNA. A single cDNA synthesis was performed on the L1-1 sample to give the L1-1/1. Two independent cDNA syntheses were performed on the R1-1 and R2-1 RNAs (giving R1-1/1, R1-1/2 cDNA samples for R1-1 RNA and R2-1/1, R2-1/2 cDNA samples for R2-1 RNA; Figure 2) to analyse reproducibility of cDNA synthesis. For all samples (L1-1, R1-1 and R2-1), 423 ng total RNA was used to make cDNA samples. First strand cDNA synthesis was performed using the SuperScript^® ^VILO™ cDNA Synthesis Kit (Invitrogen) following the manufacturer's instructions in a final volume of 20 μl. The VILO™ reaction mix includes random primers to make cDNAs. The final cDNA products were diluted 10-fold prior to use in real-time PCR.

### Primer design, verification of selected gene amplicons and gene-specific PCR amplification efficiency

For all genes, primer pairs (Table 1) were designed using Primer Express version 2.0.0 (Applied Biosystems). To check the specificity of all primers, PCR was performed on cDNA, and PCR products were analysed on a 2% agarose gel (Figure 1a). In order to confirm the sequences of the amplicons, PCR was performed on cDNA for all designed primer pairs. Each reaction contained 300 nM of each primer, 2 U of *Pfu *DNA polymerase (Promega), 400 μM dNTPs (Invitrogen), and 100 ng of cDNA in a total volume of 50 μl. Amplifications were performed with the following program: 95°C for 2 min and 35 cycles at 94°C for 30 sec, 58°C for 30 sec, and 72°C for 30 sec. PCR products were purified using the Qiaquick PCR purification kit (Qiagen) according to the manufacturer's instructions. PCR products were cloned using the TOPO TA Cloning Kit (Invitrogen) according to the manufacturer's instructions. Sequencing reactions were performed on 500 ng respective purified plasmids for each gene using 100 nM of either M13 forward or M13 reverse primer in separate reaction, 4 μl of Big Dye Mix (Big Dye Terminator v1.1 cycle sequencing kit; Applied Biosystems) and 2 μl of sequencing buffer in a total volume of 20 μl. The reactions were analysed on the ABI PRISM 3130 Genetic Analyzer (Applied Biosystems). Derived sequences for each gene were compared to the respective sequences used to design primers with Vector NTI Advance™ 10 (Invitrogen). Purified plasmids containing the respective cloned amplicon for each gene were linearised using EcoRV restriction enzyme, separated on an agarose gel, excised and purified using Sigma Gel Extraction Kit following the manufacturer's instructions (Sigma). Standard dilutions were constructed to determine the specific PCR amplification efficiency for each gene, using 10-fold of five dilution series of the purified fragments in 50 ng/μl yeast tRNA (Invitrogen). The PCR amplification efficiency of each primer pair is calculated from the slope of a standard curve as follows: for each gene, a standard curve is obtained using a 10-fold dilution series of the respective verified cloned amplicons, spanning five orders of magnitude. Based on the Cp values for all dilution points in a series, a standard curve was generated using linear regression and the slope. qBase calculates the gene specific PCR amplification efficiency using the following equation: Efficiency % = (10^(-1/slope) ^- 1) × 100% [38].

### Real-time PCR

Real-time PCR amplification reactions were performed in 384-well plates in a Lightcycler480 (Roche). Each reaction contained 2.5 μl 10-fold diluted cDNA template, 300 nM of each primer, and 1× LightCycler^® ^480 SYBR Green I Master (Roche), in a final volume of 10 μl. All reactions were carried out in duplicate for each cDNA sample. As a control for genomic DNA contamination, an equivalent amount of total RNA without reverse transcription was tested for each sample per gene. A no-template control (NTC) was also included in each run for each gene. This experiment was repeated two times in independent runs for all selected genes per plant per tissue. The thermal profile of the reaction was 95°C for 5 min activation and denaturation, followed by 45 cycles of 95°C for 10 sec, and 59°C for 10 sec. Finally, a dissociation curve was generated by increasing temperature starting from 65 to 95°C to determine the specificity of the reactions. The crossing cycle number (Cp) was automatically determined for each reaction by the LightCycler480 SW 1.5 software with default parameters using the second derivative method.

### Determination of reference gene expression stability using geNorm, NormFinder and BestKeeper

#### a) geNorm

Cp values of all samples were exported to Excel, ordered for use in qBase software and transformed to relative quantities using the gene-specific PCR amplification efficiency [49]. These relative quantities were then exported to geNorm (version 3.5) to analyse gene expression stability. The approach of reference gene selection implemented in geNorm relies on the principle that the expression ratio of two ideal reference genes should be identical in all samples, independent of the treatment, condition, or tissue type. Increasing variations in the expression ratio between two genes correspond to lower expression stability across samples. geNorm determines the level of pairwise variation for each reference gene with all other reference genes as the standard deviation of the logarithmically transformed expression ratios. In this way, the reference gene expression stability measure (*M *value) is calculated as the average pairwise variation of a particular gene with all other control genes included in the analysis [7]. Low *M *values characterise genes with the most stable expression. Sequential elimination of the least stable gene (highest *M *value) generates a ranking of genes according to their *M *values and results in the identification of the genes with the most stable expression in the samples under analysis. geNorm was also used to estimate the normalisation factor (NF_*n*_) using *n *multiple reference genes, by calculating the geometric mean of the expression levels of the *n *best reference genes [7]. The optimisation of the number of reference genes starts with the inclusion of the two genes with the lowest *M *value, and continues by sequentially adding genes with increasing values of *M*. Thus, geNorm calculates the pairwise variation V_*n*_/V_*n*+1 _between two sequential normalisation factors NF_*n *_and NF_*n*+1 _containing an increasing number of reference genes [7,36]. A large variation means that the added gene has a significant effect on the normalisation and should preferably be included for calculation of a reliable normalisation factor. Ideally, extra reference genes are included until the variation V_*n*_/V_*n*+1 _drops below a given threshold. Vandesompele and colleagues recommended a threshold of 0.15, although this threshold should not be taken as too strict of a cut-off [36].

#### b) NormFinder

For each gene, the average Cp value of each duplicate reaction was converted to relative quantity data as described for geNorm, to calculate the stability value with NormFinder program [50]. The NormFinder reference tool was applied to rank the candidate reference gene expression stability for all samples with no subgroup determination. According to the analysis, the lowest stability value will be top ranked.

#### c) BestKeeper

The average Cp value of each duplicate reaction is used (without conversion to relative quantity) to analyse the stability value of studied genes [41]. BestKeeper creates a pairwise correlation coefficient between each gene and the BestKeeper index. This index is the geometric mean of the Cp values of all candidate reference genes grouped together. BestKeeper also calculates standard deviation (S.D) of the Cp values between the whole data set. The gene with the highest coefficient of correlation with the BI indicates the highest stability.

### qBase analysis

First the acquired Cps for each gene from LightCycler for all samples were exported to Excel, then ordered for use in qBase software [49] and imported to qBase. The relative gene expression analysis of the target gene was measured using gene-specific efficiency acquired from dilution series and selected reference genes for normalisation [49]. qBase performs relative quantification using a modified delta-Ct method with the possibility to adjust for PCR efficiency and to use multiple reference genes for normalisation. The algorithm of qBase for calculation of relative quantities selecting different reference genes and specific efficiencies has four steps: 1) calculation of the average Cp value for all replicates of the same gene/sample combination within a given run, 2) transformation of mean Cp value into relative quantity using the gene specific PCR efficiency, 3) calculation of the normalisation factor and 4) calculation of the normalised relative quantity for gene of interest for each sample [49]. The relative expression of target genes for all samples can be collected from results in the qBase menu bar.

## List of abbreviations

qRT-PCR: quantitative real-time reverse transcriptase polymerase chain reaction; *ACT: *actin; *TUB*: β-tubulin; *GADPH*: glyceraldehyde-3-phosphate- dehydrogenase; *H3*: histone H3; *EF*: elongation factor 1-alpha; *rRNA*: 18S rRNA; *NADHD*: nicotinamide adenine dinucleotide dehydrogenase; 1-*FEHII*: fructan 1-exohydrolaseII; NTC: no-template control; Cp: crossing point cycle number; Tm: melting temperature; R^2^: correlation coefficient; S.D: standard deviation; BI: BestKeeper Index; *TUA*: alpha-tubulin; *UBQ*: ubiquitin; *G6PD: *glucose-6-phosphate dehydrogenase.

## Authors' contributions

AM performed all the experimental procedures, data analysis, and drafted the manuscript. EVB and MDL discussed the paper and assisted with manuscript revision. All authors read and approved the final manuscript.

## Supplementary Material

Additional file 1 **A partial segment alignment of *****FEHIIa*****and *****FEHIIb *****amplicon used for qRT-PCR and position of exon andintron in*****FEHIIa*****gene.** Click here for file
