# Supplementary material for: Validation of reference genes for gene expression analysis in chicory (Cichorium intybus) using quantitative real-time PCR
Source: BMC Mol Biol. 2010 Feb 15;11:15. doi: 10.1186/1471-2199-11-15 (PMC2830926; doi:10.1186/1471-2199-11-15)
Supplement: Additional file 1 — A partial segment alignment of FEHIIaand FEHIIb amplicon used for qRT-PCR and position of exon andintron inFEHIIagene. [file 1471-2199-11-15-S1.PDF]

a

1 CCGCAGACCTCTATCCATATATTAGTTCTTTTTCTCCATATGTTGTCGTGCTGTCCAAAC
61 AGTCACTTTTCGCTTTTTGAATAGTTATATCTTTTATCATTGCAATGAATGATATTTTTG
121 ATCAATAATATCAATTATCTGTATTAAATAGATATTTTTAGTTTTATATGCTGTATAATT
181 ACTATGTTTGTCCAATAATGCTAGCTTTTACATCTCCAATTACGTTTTTACCATCAAAGA
241 ATTATTATTTTTTGATTTCAGATTTTGTAGTATATATTTTAATTTCGTCTATGGTAATAT
301 ATTGATTTTATTTAATGTTATTGTTAACTAGTTTTTAATCTTATATATTCTAGATGTCCT
361 TAAAAATAAAAAATTAAATGATGTTTTATAAAATATTTCATAAATATGAGGACAATTAAT
421 TAACTTAAACAACAAATTAGTGGTCAATGGTTTGTTTAATAAATTTAGTGGAAATGTTAC
481 ATAGAAAAAACATGGCTCATGAGCCCTGTGATCGTGAATGTTCTATTACTTTTCTATTCA
541 GAACGTGCAACATGGACTAGTAGTTTCTTTCAAAACAACGTAAAAGTATAAAACTATTGA
601 TTTATCCATAAATAAAAATTGCGCTCGTATATGTAAAAGAAACTACAAACTCATAAATGA
661 ATATGCCGTCATTTACTTATTAATACTTATAAATGTACTGGTAATGAGATAGAAGTAAAA
721 TGTTTATCAAATGTCAATAGAGAAAAACTCTATACAACTCAAACTAAAACGGATGTATTT
781 TTAAATAGACCATATTATACATCTTTACAAAGAAAAACATAATTTAGAAGTTAGTCCTTT
841 GTTACGGCGGCTAGATATTTCAAAGTCGTAAAAAATGGGTCCTTTAAAGACAATGAAATA
901 AAAAATACATAATCGAGGATGACACGTATATTAATAGTAAAAAGTGAGGGGTAGGCCTGG
961 CCAACTTTTCAAAATAACGAAAGTTCAAGGGGCAATAAGTCAATAACTAACATAATTCGG
1021 TAGATACTATTTGTCTCAAAGTCTATAAAACCAACCAATGGACCATTCTCAATTCTCATT
1081 CCATCCATAACAACACCCATTCTCCTCAACAACATTAAAGTCATCGATCCCCAAACACAC
1141 ACACTCATCATGAAGAAATCACTTTCTTCATTTATTGTATTATGTTTTCTTGTCATCATT
1201 CTGGAAACTGGTCGGGTAAAAGCGACCAGTCGGAATCTGAATGATGTGATAATGCTGGCG
1261 AATCAGCAGATTGAACAGCCGTATAGAACTGGATACCATTTTCAACCTCCAAGCAACTGG
1321 ATGAACGGTACACCTACCTTTTTCTTTTATTAGAATCTTTATTTATATATTTTGAAGAGA
1381 ATTATTTTTGAAATTATGGTATTTGAATTTTTAATGCATATAAACTGTCTGCATGGATAA
1441 TCAATCAATTTATGTGAAATTGAAAATTTTAAAAATTCGATTAAAGATTTCTTTTCATTG
1501 ATGTTTTATGGTAGCTAACTTTTTTTTTTTCTTTTGGTGTTCCATTGACATGGATACAAAA
1561 TGGACACGATGGTTGGTTGGAATTTCGCAGATCCCAATGGTGAGTTTTATAATTTTGGAC
1621 ATTTTTATTTTTTTAATTTAAGAAAAACGGTTGTTAAATCTTGAAGTAACTGCCAATGTT
1681 ATTTAATATTATTATAATGGGCAATGAAAATAATATCATCGTAAAAAACAAGAAAAATGC
1741 ATCCAAGGTTATTTATTACTTTAAGCATAAAAATGACTTTTATCTTAATAATATAATAAA
1801 AGGAAAGTCACAGCTTAATTTGAATGGATAGAGTGACGAGGAAAAAAGGAACCCTAACAA
1861 ATACGCAGAAGGAGAATCCCTTTTCCCCCCGGCCAGCCCCTTTTTTTTCGATATTTATGA
1921 CATATAGCCCCTATAGCTTTTCTTTTTAGCTTTTTTCCTATCCCTTATTCGTTTTTATGA
1981 TTTTTATTAAAAAATTATATATGGATAAACCAGTAATTGGAAGTGAATACGGTTTATTTT
2041 GTGGCGAAGATTTTATATTTAATTGAAAGCATAATCTACCAAATATATAGTAAAATACAA
2101 GGAAAAAAGTTGAAAAAATATAATTTGCAAAAAAAAAAGCTCTTTATATATACATAGAAA
2161 TATGCAAAATAAGCACGGAATAAATTCTTGAAAATTTAAATTTTAAAATAAAATATTAAA
2221 AAACTGAAAAAGCAATATTAAAATAAGTAAGTTTTTCAAACCATACAAAAAAATTAACTT
2281 TAGCAAAATTGTAAGCGAAAAATTAGGTTTGAAAATTAAATTGTCAAAAAGATTCACTAG
2341 ATAAACATATAATATATCATTAAAGTCGAGAAAATGATTTCACAATCTTAATCAAAATAA
2401 TAAATGAAAAATATTATGCTTTATATAAATATGTGTATTTGGATGGATTAGATTTCGGAT
2461 AACATCGATAAGTTACTTGGTTGGACCAGGTCCTTTAGGATTGGTCGGCACCAATCATAA
2521 CATATTTTTCTATGTATTTGACTAAATACGAATATTTTATTCATAGGTATTTCTCTAAAC
2581 TACTAATAATTAAATATCATCGAGTTTCAACAAATTTAAATTGTCATTTAAAATTTAAAA
2641 CTCAAAGGATATAATAATCATTTTGATAAATTTTTTTATTGTATTTAGGACCAATGTTAT
2701 ACCAGGGAGTGTACCATTTCTTCTACCAATACAATCCGTATGCAGCAACGTTTGGTGACG
2761 TCATAATCTGGGGCCACGCCGTATCATACGACTTAGTCAACTGGATCCATCTTGACCCGG
2821 CAATTTACCCGACCCAAGAAGCTGACAGCAAGAGTTGCTGGTCGGGATCCGCCACCATCC
2881 TACCGGGGAATATTCCGGCCATGTTGTACACCGGCAGCGATTCAAAGTCCCGTCAAGTAC
2941 AAGACCTTGCCTGGCCCAAAAACCTCTCCGACCCGTTCCTTCGTGAATGGGTGAAACACC
3001 CGAAAAACCCACTCATAACCCCACCGGAGGGCGTTAAAGACGACTGTTTCCGTGACCCGA
3061 GCACCGCCTGGCTCGGCCCCGACGGCGTATGGAGGATCGTCGTCGGCGGAGACCGTGACA
3121 ACAACGGTATGGCGTTTTTATACCAAAGTACCGATTTCGTCAACTGGAAACGATACGACC
3181 AGCCTCTTTCGTCGGCGGATGCCACCGGAACTTGGGAGTGCCCGGACTTTTACCCTGTGC
3241 CGTTGAACAGCACCAACGGGCTCGATACGTCGGTGTACGGCGGCAGTGTGAGACATGTAA
3301 TGAAAGCAGGATTTGAAGGGCATGATTGGTACACAATTGGGACTTACAGTCCTGATCGTG
3361 AAAACTTTTTGCCGCAAAATGGGTTGAGTTTGACCGGAAGTACGTTGGATTTGAGGTACG
3421 ATTATGGCCAATTTTATGCTTCCAAATCGTTCTTCGATGATGCCAAGAACAGAAGGGTTT
3481 TGTGGGCGTGGGTTCCTGAAACTGATTCTCAAGCAGATGATATTGAAAAAGGATGGGCTG
3541 GTCTTCAGGTAGTGTTTTAGATTTAATTTTTAGAAAATAATATAAACCCAATTTATAATA
3601 TTTATGAATATAAAAAAAGAACATTTACGCTTAATAATGGGTTAATTTTATAAAAGTCAC

```
3661    TTTTGGTTTTTTTGTTTTAATCATGGAAAGAATTTTTTTTTAATTTAACTATCAAAAGTT
3721    TGTTTAACCATTTAAACAATTGATTTTTAATATAAACAATTCCAAAATTTGAACGTTTTC
3781    CAAATAAGTCTCAACTATCTAGATTAATTATTATTCCAAAAGGTTTTTGTAACTCATTAT
3841    TGGAAGTAAAAAAAGTATAATGGTTATTTTGAAAAGAAAAACAAATCTACAGTGGTTTAT
3901    TTAAAAATAAAAACAAACTTTCAATAATTAAACTTAAAAAAATTGATAAACCTAAAAAAA
3961    TCAAACTATAATTGTGATAAATAAAATTAATCCTTAATAATTTGGTTCACACTTAATTTC
4021    TTTTTCTAATTGTAGTCATTCCCAAGAGCACTTTGGATCGACAGAAATGGGAAACAGTTG
4081    ATTCAATGGCCTGTTGAGGAGATTGAAGAACTTCGTCAAAATCAAGTCAACCTTCAAAAC
4141    AAAAACCTTAAACCCGGTTCGGTTCTTGAAATCCATGGGATTGCTGCTTCTCAGGTTCGT
4201    ACCTTTTGCTTAAAAAGTTGTATTTTCTTCAGAGTTTGTACTGAAATGTTTTATTTCGGA
4261    AATTTAGGCGGATGTGACAATTTCGTTCAAATTGGAGGGGTTGAAAGAGGCAGAGGTTTT
4321    GGATACGACGTTGGTTGATCCACAAGCGCTTTGTAACGAAAGAGGTGCTTCAAGTCGTGG
4381    TGCTCTTGGGCCTTTTGGCTTGTTGGCTATGGCGTCTAAAGACTTGAAAGAACAAAGTGC

4441    AATCTTTTTTAGGGTTTTTCAAAACCAACTTGGACGATACTCTGTGCTCATGTGTAGTGA
4501    TCTTAGCAGGTTTGTGGTTATTCATTTATTTGTTGTACAAACACAATATAAATGTGGGTT
4561    GATTGTGAAAAAAGTATTATATTTTTGCCAATCGTTCGGTTTAACCATTAACTTTTTTAA
4621    AATTTGGAAAAAAATTAATCAATTTTTTGTAAAAAGTTTCATCGAAGTCATTAGTACCGG
4681    TTATTCCGGTCATAATAGTTGAGCTAAAACTTTTTTTAATTTAAATTTGGTGGATCATTA
4741    AAACAACTGAAAATTTGATGGTTAAATTAGAAAAAATGATGAAATATAGTGTTTTTTTCG
4801    TAATTATATAAATTAAAGCGTAAATATTATCTAGGACTAATTACGAAAAACACTATATTT
4861    TGTCAAAATTCAGTTTAACCATCAATTTTTAAGATTAAAAAAAACACTTTGTTTTANTTT
4921    TAAGAATTAAAAAAAAACCCTTTTGTTTTTATTAAAAGTTTCATCGAATGCATTCTTACC
4981    GACTTNTTCAGGTCCCCAAGTCGGGGTTAAAATTTTTTCCCAAAAATTGATGGCTTTATT
5041    AAAAACCCCAAAAAAAATTTTAATGATTAAACCAGAAAAAATCTAAAATATAATATTTTTT
5101    CGTAATTGAACATGCTACATATTTTGTGTATGAAATAGTTTCCAACAATTAATTGTTTTT
5161    TTATTCTTGTATAAAAGGTCTACTGTTAGAAGCAATATCGACACGACAAGTTATGGTGCA

5221    TTTGTTGACATAGATCCTCGGTCTGAGGAGATCTCGCTAAGAAACTTGGTATGGAATGTT
5281    TTATTTAGTGTCATGGATGCCCTCGAAATTTCCATCTTTACACTTTTGACCCCTTGTTTG
5341    TTGAATGTTTATAGATTGACCACTCAATTATTGAGAGTTTCGGAGCTGGTGGGAAAACAT
5401    GTATCACAAGTCGGATTTACCCAAAATTTGTTAACAATGAAGAGGCTCATCTTTTTGTGT
5461    TCAATAATGGGACTCAAAATGTCAAAATTTCGGAGATGAGTGCTTGGAGTATGAAGAATG
5521    CTAAATTTGTTGTTGATCAGAGTGTAAAAAGTGCAGCATAAAAAAAATAGAGGGCAAGAA
5581    TGTTAAAAATGTAAAAAGTGCAGCATAAAAAAAATAGAGGGCAAGAATGTTAAAAATAGG
5641    TAGAGTATTCTTGTTATAAAGTAAAGTAAAGGAACCAGTGAAGGTTGAAAGAGTAGGTTT
5701    GGTAGCTCTTTCGTAAAGATCATTCTTTGTTGCTGTATTCCTTGTGTAAAAATTTTGAGG
5761    TTGTATCGATTTTTTATGCCTTTGTAAGTTATTGAGATGTAAGATTTGTGCAAAGCATTG
5821    TAACAAATTATTGTTTCATTAATACAACTACAATATTTGAGC
```

b

```
FEHIIa 4417   TAAAGACTTGAAAGAACAAAGTGCAATCTTTTTTAGGGTTTTTCAAAACCAACTTGGACG   4476
              ||||||||||||||||||||||||||||||||||||||||||||||||||||||||| ||
FEHIIb 1356   TAAAGACTTGAAAGAACAAAGTGCAATCTTTTTTAGGGTTTTTCAAAACCAACTTGGGCG   1415

FEHIIa 4477   ATACTCTGTGCTCATGTGTAGTGATCTTAGCAGGTCTACTGTTAGAAGCAATATCGACAC   4536
              ||| ||||| ||||||||||||||||||||||||||||| ||||| ||||| ||||||||
FEHIIb 1416   ATATTCTGTTCTCATGTGTAGTGATCTTAGCAGGTCTACCGTTAGGAGCAACATCGACAC   1475

FEHIIa 4537   GACAAGTTATGGTGC   4551
              |||||||||||||||
FEHIIb 1476   GACAAGTTATGGTGC   1490
```

(a) Full sequence of *FEHIIa*. Red indicates the promoter. Black arrows represent the primer pair for the *FEHIIa* amplicon. (b) *FEHIIa* and *FEHIIb* RT-PCR amplicon, green indicates exon 5 and blue indicates exon 6 in the *FEHIIa* gene. The numbers indicate the reference base pairs, bases 1 to 5862 for *FEHIIa*; GenBank accession number AY323935 for *FEHIIa* full gene sequence and AJ295034.1 for *FEHIIb* mRNA.
